# Expression of SATB1 Promotes the Growth and Metastasis of Colorectal Cancer

**DOI:** 10.1371/journal.pone.0100413

**Published:** 2014-06-27

**Authors:** Yi Zhang, Xiuyun Tian, Hong Ji, Xiaoya Guan, Wei Xu, Bin Dong, Min Zhao, Meng Wei, Chunxiang Ye, Yuan Sun, Xiaosun Yuan, Chen Yang, Chunyi Hao

**Affiliations:** 1 Key Laboratory of Carcinogenesis and Translational Research (Ministry of Education), Department of Hepato-Pancreato-Biliary Surgery, Peking University School of Oncology, Peking University Cancer Hospital and Institute, Beijing, People’s Republic of China; 2 Department of Gereology, Aerospace Center Hospital, Beijing, People’s Republic of China; 3 Key Laboratory of Carcinogenesis and Translational Research (Ministry of Education), Center Laboratory, Peking University School of Oncology, Peking University Cancer Hospital and Institute, Beijing, People’s Republic of China; 4 Key Laboratory of Carcinogenesis and Translational Research (Ministry of Education), Etiology Laboratory, Peking University School of Oncology, Peking University Cancer Hospital and Institute, Beijing, People’s Republic of China; National Cancer Center, Japan

## Abstract

Special AT-rich sequence-binding protein-1 (SATB1) has been identified as a genome organizer that reprograms chromatin organization and transcription profiles. SATB1 promotes tumor growth and metastasis in breast cancer and is associated with poor prognosis in several cancer types. The association between SATB1 and colorectal cancer (CRC) has not been studied intensively. Therefore, this study aimed to investigate the effect of SATB1 on CRC growth and metastasis in vitro and in vivo and its correlation with overall survival and clinicopathological factors in CRC patients. Stable SATB1 knockdown and SATB1-overexpressing cell lines were established. SATB1 knockdown decreased cell growth, colony formation, migration, and invasion and increased apoptosis in CRC cells in vitro (p<0.05), whereas SATB1 overexpression had the opposite effect. SATB1 overexpression increased tumor growth and metastasis to lung and liver in vivo by using xenograft animal models (p<0.05). Thus, SATB1 promoted an aggressive CRC phenotype in vitro and in vivo. Immunohistochemical analysis of 560 CRC specimens showed that SATB1 expression was significantly higher in CRC tissues than in matched non-tumor mucosa (p<0.001). In addition, SATB1 expression was significantly higher in patients with poorly differentiated tumors, higher invasion depth, distant metastasis, and advanced TNM stage. SATB1-positive patients had a poorer prognosis than SATB1-negative patients, and SATB1 was identified as an independent prognostic factor for CRC (p = 0.009). Strikingly, we also evaluated SATB2 expression in CRC and found that SATB2 was more abundantly expressed in non-cancerous mucosa compared to colorectal cancer tissues (p<0.001). However, SATB2 expression had no influence on prognosis of CRC patients (p = 0.836). SATB1 expression was significantly associated with shorter survival time either in SATB2-positive patients or in SATB2-negative patients (p<0.001). In conclusion, our findings indicated an important role for SATB1 in CRC tumorigenesis and metastasis. Therefore, SATB1 may represent an important prognostic biomarker and therapeutic target for CRC.

## Introduction

Colorectal cancer (CRC) is the third leading cause of cancer-associated death in the United States of America [1] and the second most prevalent cancer in China [2]. Approximately 15–25% of CRC patients experience synchronous liver metastases, and 80–90% of these patients have unresectable metastatic liver disease [3]. Metastatic liver disease is the major cause of death in CRC patients [4]. Therefore, there is an urgent need to identify sensitive and specific molecular markers to predict CRC metastasis. Further understanding of the underlying mechanisms of CRC metastasis is essential in the identification of biomarkers for metastatic progression in CRC.

Special AT-rich sequence-binding protein-1 (SATB1) is a tissue-specific nuclear protein that is predominantly expressed in thymocytes [5] and was originally recognized for its critical role in proper T-cell development [6]–[8]. SATB1 binds special AT-rich anchor sites circumscribing heterochromatin to form a “cage-like” functional nuclear architecture that serves as a landing platform for chromatin-remodeling factors. Therefore, the SATB1 network may regulate gene expression by altering the functional organization of DNA sequence [9], [10]. SATB1 has been recently reported to be a genome organizer. SATB1 expression markedly altered the expression of over 1000 breast cancer genes including metastasis-associated genes and tumor suppressor genes to promote growth and metastasis of breast tumor [11]. Furthermore, multivariate survival analysis showed that SATB1 was an independent prognostic factor for breast cancer [11]. SATB1 overexpression has also been associated with poor prognosis in laryngeal squamous cell carcinoma [12], gastric cancer [13], [14], and malignant cutaneous melanoma [15]. The association between SATB1 and colorectal cancer (CRC) remains unclear.

In this study, we demonstrated the involvement of SATB1 in CRC growth and metastasis based on the following evidence: (a) SATB1 overexpression was detected in both CRC cell lines and CRC tumors, (b) growth and colony formation rates were down regulated in SATB1-knockdown cells but up regulated in SATB1-overexpressing cells, (c) migration and invasion capabilities were much poorer in SATB1-knockdown cells, whereas more aggressive in SATB1-overexpressing cells, (d) SATB1 overexpression promoted carcinogenesis and metastasis in vivo by using animal models, (e) the expression of SATB1 protein was more abundant in CRC tissues than in matched non-cancerous tissues, and (f) SATB1 expression was found to be an independent prognostic factor for CRC patients.

## Materials and Methods

### 2.1 Cell Lines and Cell Culture

SW480, SW620, HT-29, HCT116, RKO, and LoVo CRC cell lines were purchased from American Type Culture Collection (ATCC) and Chinese Academy Of Medical Sciences & Peking Union Medical College, and all the cell lines were maintained in Dulbecco’s modified Eagle’s medium (DMEM; GibcoBRL, Life Technologies, Grand Island, NY, USA) supplemented with 10% fetal bovine serum (FBS), streptomycin (100 µg/ml), and penicillin (100 µg/ml). All cell lines were cultured at 37°C under 5% CO_2_.

### 2.2 Establishment of Stable SATB1-knockdown Cell Lines

Three short-hairpin RNA (shRNA) sequences were designed based on the SATB1 sequence (NM_002971) identified by shRNA Target Finder (Ambion; Life Technologies, Carlsbad, California): shRNA1 (2566), 5′-GTCCACCTTGTCTTCTCTC-3′; shRNA2 (2364), 5′-AAGGACAATTCCGGTTTAGAG-3′; and shRNA3 (2797), 5′-AAAGTGTGTACCCCGTAAGCA-3′. The oligoduplexes were cloned into the pSilencer3.1 vector (Ambion; Life Technologies, Carlsbad, California). The resultant constructs were transfected into LoVo cells or RKO cells using Lipofectamine 2000 (Invitrogen, Life Technologies, Carlsbad, California) according to the manufacturer’s instructions. Cells transfected with empty vector (pSilencer3.1) were used as controls. Stable shRNA transfectants were selected by culturing with 600 µg/mL of G418 (GibcoBRL) for 3 weeks. RT-PCR, western blot, and immunofluorescence staining were used to confirm the down regulation of SATB1 expression.

### 2.3 Establishment of Stable SATB1-overexpressing Cell Lines

Full-length human SATB1 cDNA was cloned into the pcDNA3.1 expression vector (Ambion; Life Technologies, Carlsbad, California). SW480 cells were transfected with SATB1-pcDNA3.1 or empty vector (pcDNA3.1) as a control. Transfectants with stable SATB1 overexpression were selected by culturing with 600 µg/mL of G418 for 3 weeks. RT-PCR, western blot, and immunofluorescence staining were used to confirm the up regulation of SATB1 expression.

### 2.4 Patients and Samples

CRC tumor tissues were obtained from 560 patients who were diagnosed and treated in Department of Surgery, Peking University School of Oncology between 2005 and 2008. None of the patients received chemotherapy or radiation therapy before surgery. Five-hundred and forty-two of these patients were followed-up postoperatively, over a period of at least 3 years. And detailed clinicopathological information was obtained from 520 patients. Fresh colorectal tumor and matched normal colorectal mucosa specimens were obtained from 21 patients. Specimens were snap frozen in liquid nitrogen and stored at −80°C until analysis. Histopathological analyses were performed independently by two pathologists. For the use of clinical materials for research purposes, approval from the Regional Ethical Committees, Beijing Cancer Hospital, China was obtained. Written informed consents have been obtained from all participants. The clinical investigation was conducted according to the principles expressed in the Declaration of Helsinki.

### 2.5 Extraction of Total RNA and Reverse Transcription-Polymerase Chain Reaction

SATB1 mRNA expression was determined by reverse transcription-polymerase chain reaction (RT-PCR). Total RNA was extracted using Trizol reagent (Invitrogen, Life Technologies, Carlsbad, California) according to the manufacturer’s instructions. cDNA was synthesized from the extracted RNA (4 µg) using the EasyScript First-Strand cDNA Synthesis SuperMix kit (Invitrogen). PCR amplification was performed using the following SATB1 and beta-actin gene-specific primers: SATB1, 5′-AGAGGAAGGCTTGGGAGTA-3′ (sense) and 5′-GGGCAGCAGAGCTATGTG-3′ (antisense); and Beta-actin, 5′-TTAGTTGCGTTACACCCTTTC-3′ (sense) and 5′-ACCTTCACCGTTCCAGTTT-3′ (antisense). Beta-actin was used to normalize SATB1 gene expression. Twenty-eight PCR cycles were performed in which each cycle consisted of pre-denaturation at 94°C for 3 min, denaturation at 94°C for 45 s, annealing at 55°C for 45 s and extension at 72°C for 45 s, and after the 28 cycles is a final elongation at 72°C for 10 min. PCR products were separated by gel electrophoresis on 2% agarose gel stained with ethidium bromide. Band intensity was measured directly on an Alpha Imager 2200 analysis system (Alpha Innotech, San Leandro, CA, USA).

### 2.6 Western Blot Analysis

Cells and fresh tissues were lysed in 1×sodium dodecyl sulfate (SDS) lysis buffer. Equal amounts of total protein were separated by SDS-PAGE, electrotransferred onto polyvinylidene difluoride membranes (Pall Corporation, Mexico City, Mexico), and incubated overnight at 4°C with a SATB1 rabbit polyclonal antibody (1∶500 dilution, PRS4631; Sigma-Aldrich, St. Louis, MO, USA). Beta-actin (1∶10000, Sigma-Aldrich) was used as a loading control. Protein bands were evaluated by enhanced chemiluminescence (Thermo Fisher Scientific, RockFord, IL, USA).

### 2.7 Immunofluorescence Staining and Laser-scanning Confocal Microscopy

Cells were cultured on glass coverslips, fixed with 4% paraformaldehyde, permeabilized using 0.1% Triton X-100, washed, and blocked with 5% bovine serum albumin. The cells were incubated with SATB1 primary antibody (1∶100, PRS4631, Sigma-Aldrich) followed by incubation with a rhodamine-conjugated secondary antibody (1∶50; Zhongshan Golden Bridge Biotechnology, Beijing, China). Coverslips were mounted with vectashield mounting medium (Vector Laboratories, Burlingame, CA, USA) containing diamidino-2-phenylindole (DAPI) nuclear stain and examined under a fluorescence microscope, then the images were constructed using a software (LAS AF 2.2.1 version, TCSSP5, Leica, Germany).

### 2.8 Cell Growth

Cell growth was evaluated by 3-(4.5-methylthiozol-2yl)-2.5-diphenyltetrazolium bromide (MTT) assay. Cells (3×10^3^ cells/well) were seeded into 96-well plates and cultured for 24, 48, 72, and 96 h. MTT (10 µL) was added into each well, and cells were cultured for an additional 4 h. The culture media was removed and 200 µL DMSO (AMRESCO Inc., Solon, OH, USA) was added to each well. The absorbance at 570 nm was measured with a microplate reader (iMark, Bio-Rad Laboratories, Hercules, CA, USA). Cell growth curves were constructed by plotting absorbance (blanked by DMSO) against time.

### 2.9 Apoptosis and Flow Cytometry Analysis

Stable SATB1-overexpressing, SATB1-knockdown, and empty vector control cells (1×10^6^ cells) were cultured in 60-mm dishes for 48 h and harvested. The cells were labeled with propidium iodide and AnnexinV. Negative controls consisted of unlabeled cells and appropriate isotype controls. A minimum of 10,000 events for each sample were collected and analyzed using a flow cytometer (FACSAria; BD Biosciences, San Jose, CA, USA).

### 2.10 Cell Cycle Analysis by Flow Cytometry

Stable SATB1-overexpressing, SATB1-knockdown, and empty vector control cells were cultured in 60-mm dishes and serum starved for 24 h. Cells were then cultured in DMEM supplemented with 10% FBS for 48 h. Viable cells were harvested, fixed in 70% ethanol and analyzed by flow cytometry.

### 2.11 Soft Agar Colony Formation

Stable SATB1-overexpressing, SATB1-knockdown, and empty vector control cells (3×10^3^) were resuspended in DMEM containing 3 mL 0.4% agarose, 20% FBS, 0.5 mM sodium pyruvate, 10 m MHEPES, and 1% penicillin/streptomycin and layered on top of 4 mL of 0.8% agarose in DMEM on 60-mm dishes. After 3 weeks, colonies were counted and photographed.

### 2.12 Wound Healing Assay

Stable SATB1-overexpressing, SATB1-knockdown, and empty vector control cells were cultured in 60-mm dishes until the cell density reached 90% confluence. A horizontal wound was created using a sterile 10-µL microtip. The cells were washed with phosphate-buffered saline (PBS) to remove cell debris. Three different areas in each dish were selected to compare the distance of migrating cells from the wound origin. Images were captured at 0 and 24 h to assess wound closure.

### 2.13 Transwell Migration and Invasion Assays

Stable SATB1-overexpressing, SATB1-knockdown, and empty vector control cells (1×10^5^) were suspended in 200 µL serum-free medium and seeded into the upper chamber of a transwell insert with an 8-µm pore size membrane (Corning Costar Corp, Cambridge, MA, USA). DMEM containing 10% FBS was placed in the lower chamber as a chemoattractant. After incubation for 24 h, non-migrated cells in the upper chamber of the transwell insert were removed with a cotton swab, and the migrated cells on the underside of the filter membrane were fixed and stained with 0.1% crystal violet. The number of migrated cells was counted in 5 randomly selected microscopic fields and photographed. The protocol used for the invasion assay was the same as that used for the migration assay, except that the transwell insert was coated with Matrigel (BD Biosciences, Heidelberg, Germany).

### 2.14 In Vivo Carcinogenesis

Stable SATB1-overexpressing and empty vector control cells (2×10^6^) in 100 µL Hank’s balanced saline solution (HBSS; GibcoBRL, Life Technologies) were injected into 5 female 4-week-old BALB/c athymic mice. SATB1-overexpressing and empty vector control cells were injected subcutaneously into the left and right dorsal flank of each mouse, respectively. Tumor volumes were measured twice weekly, and mice were sacrificed after 4 weeks. Tumors were excised, measured, and weighed. Tumors were fixed in 10% formalin for histopathological analysis or stored at −80°C for RT-PCR and western blot. All animal experimentation was done in accordance with relevant national and international guidelines. The protocol was approved by the Committee on the Ethics of Animal Experiments of Peking University School of Oncology (Permit Number: 2012-07). All surgery was performed under sodium pentobarbital anesthesia, and all efforts were made to minimize suffering.

### 2.15 Xenograft Animal Model of Lung and Liver Metastasis

We determined the effect of SATB1 expression on CRC metastasis in xenograft mouse models of lung and liver metastasis. In the liver metastasis model, single-cell suspensions of stable SATB1-overexpressing and empty vector control cells (2×10^6^) in 100 µL HBSS were slowly injected into the spleen of BALB/c athymic mice under anesthesia. In the lung metastasis model, single-cell suspensions of SATB1-overexpressing and empty vector control cells (2×10^6^) in 100 µL HBSS were injected into the lateral tail vein. Five mice were included in each treatment group. Mice were sacrificed 8 weeks after injection, and the spleens, livers, and lungs were surgically excised. The number and size of metastatic foci in the liver and lung were documented. Specimens were fixed in 10% formalin or stored at −80°C for future analysis.

### 2.16 Immunohistochemical Analysis of Tissue Microarrays

Formalin-fixed, paraffin-embedded colorectal tumor and adjacent normal colorectal mucosa specimens were constructed into tissue microarrays (TMAs). TMA block sections (4 µm) were deparaffinized and rehydrated in graded alcohol. Antigen retrieval was performed in a microwave by EDTA buffer. Cells were incubated in 3% hydrogen peroxide for 15 min to block endogenous peroxidase activity and then in 5% non-fat milk to prevent non-specific binding. TMA block sections were incubated overnight at 4°C with a specific SATB1 rabbit monoclonal antibody (1∶50, ab92307; Abcam, Cambridge, CA, USA) or a rabbit monoclonal antibody against SATB2 (1∶200, TA307098; OriGene Technologies, Inc.). For the negative controls, primary antibodies were replaced with PBS. All sections were examined microscopically and scored by two independent pathologists who were blinded to the clinical information of the subjects. Unlike ordinary sections, SATB1 or SATB2 immunohistochemical staining on the tissue microarray chips was almost 100% nuclear-dyed or non-dyed for tumor tissue or normal colorectal mucosa, so when assessing their staining, we only took into account of staining intensity of positively stained nucleus. Nuclear intensity was denoted as negative, weakly positive, moderately positive or strongly positive. And when carrying out statistical analysis, we combined the patients of weakly positive, moderately positive and strongly positive staining as one positive-staining group.

### 2.17 Statistical Analysis

SPSS 16.0 software (SPSS Inc., Chicago, IL, USA) was used to perform the statistical analyses. All in vitro experiments were performed in triplicate and repeated 3 times. Unpaired 2-tailed *t* tests were used for group comparisons after verifying normality and homogeneity of variance. Data in the figures and text are presented as the means ± standard deviation. Two-tailed chi-squared test (χ^2^) or Fisher’s exact test was used to evaluate the relationship between SATB1 expression and clinicopathological factors or SATB2 expression. Kaplan-Meier survival analysis was used to evaluate patient prognosis, and p values were calculated by log rank test. Multivariate analysis by Cox proportional hazards regression model was used to determine the effect of SATB1 expression and clinicopathological factors on patient survival. A p value of <0.05 was considered significant.

## Results

### 3.1 Expression of SATB1 in CRC Cell Lines

**Figure 1:**
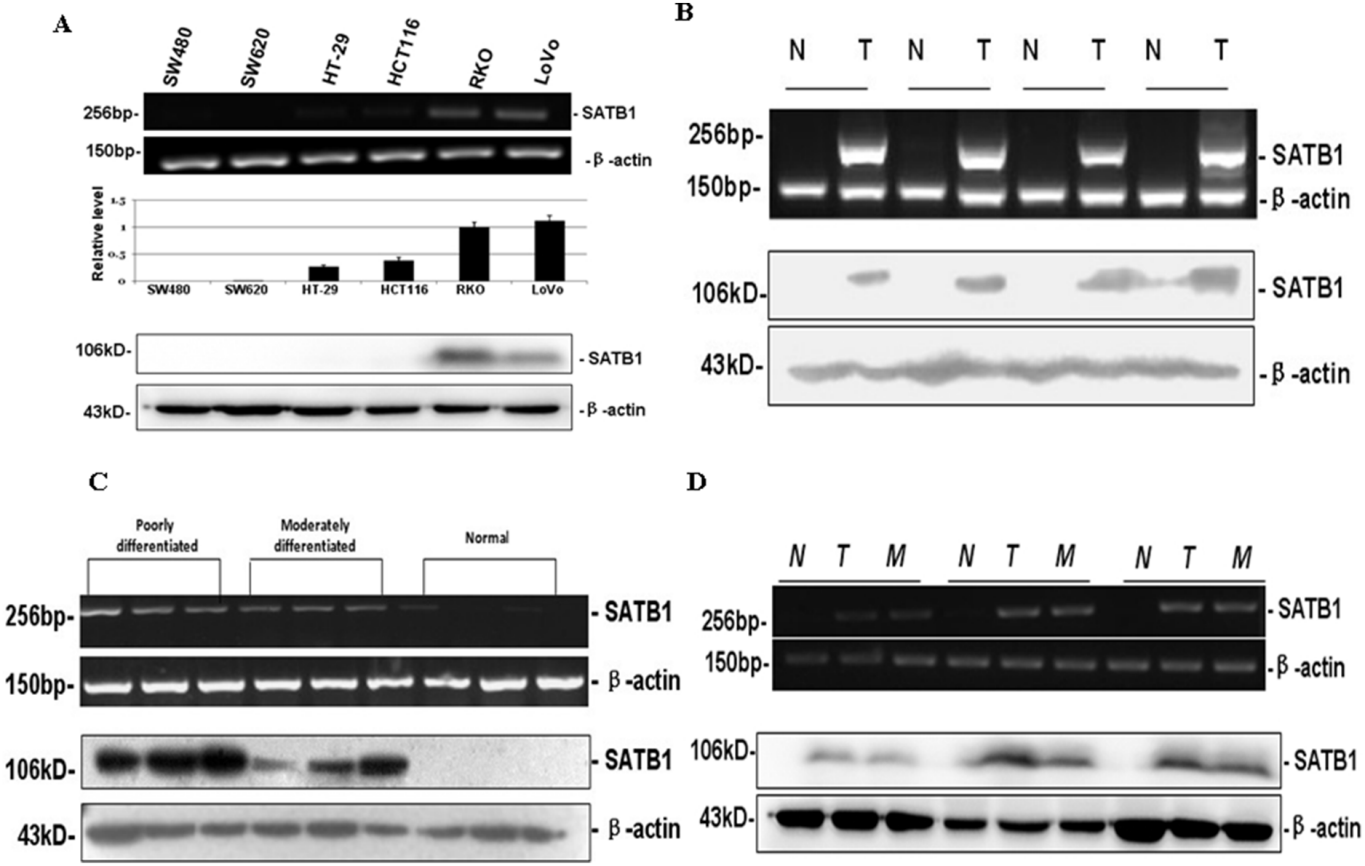
SATB1 mRNA and protein expressions in CRC cell lines and tissues. (A) SATB1 mRNA and protein expressions were determined in (A) 6 CRC cell lines, SW480, SW620, HT-29, HCT116, RKO, and LoVo; (B) representative colorectal tumors and matched normal mucosa; (C) moderately and poorly differentiated tumors and normal mucosa; and (D) representative colorectal tumors and matched synchronous hepatic metastasis foci tumors. SATB1 mRNA and protein expression were determined by RT-PCR and western blot, respectively, and normalized to β-actin. T, colorectal tumors; N, matched normal mucosa; M, synchronous hepatic metastasis foci tumors.

The mRNA and protein expression of SATB1 in 6 human CRC cell lines, SW480, SW620, HT-29, HCT116, RKO, and LoVo are shown in Fig. 1A. SATB1 mRNA and protein expression were not detected in SW480, SW620, HT-29, and HCT116 cells. SATB1 mRNA was detected in the highly metastatic RKO and LoVo cell lines. In addition, SATB1 protein was highly expressed in RKO and LoVo cells. So LoVo and RKO cell lines were chosen for a series of SATB1 knockdown experiments, and SW480 cell lines for the SATB1 overexpression experiments.

### 3.2 Expression of SATB1 in Primary CRC and Hepatic Metastasis Foci

The expression level of SATB1 mRNA and protein was determined in primary CRC tumor and matched non-tumor mucosa specimens. Among the 21 matched cases, SATB1 mRNA and protein were detected in 17 tumor samples but not in any of the non-tumor mucosa samples (Fig. 1B). Furthermore, SATB1 mRNA and protein expression were higher in poorly differentiated tumor samples than in moderately differentiated tumor samples (Fig. 1C). SATB1 mRNA and protein expression in primary CRC was similar to that in matched synchronous hepatic metastasis foci (Fig. 1D).

### 3.3 Suppression of SATB1 Expression Reduces Cell Proliferation, Colony Formation, Migration, and Invasion in Vitro

**Figure 2:**
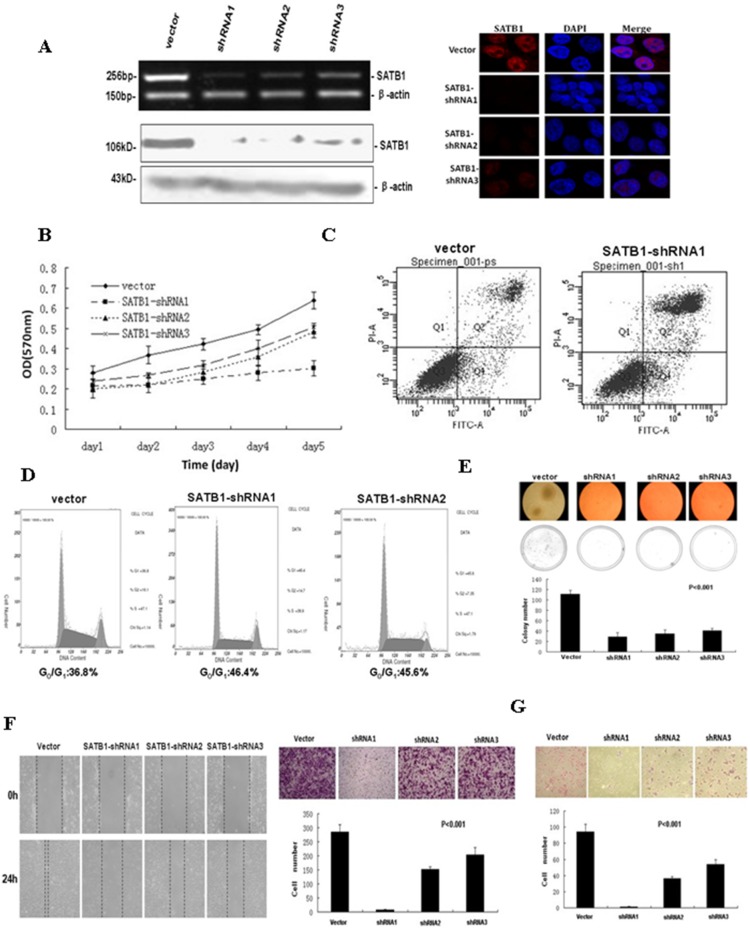
In LoVo cell lines, knockdown of SATB1 expression reduces cell proliferation, colony formation, migration, and invasion in (A) Three stable SATB1-knockdown LoVo cell lines were established using shRNA. Cells transfected with empty vector served as controls. The efficacy of SATB1 knockdown was verified by RT-PCR (top left panel), western blot (bottom left panel), and immunofluorescence (right panel). β-actin was used to ensure equal loading. (B) Cell proliferation of empty vector control, SATB1-shRNA1, SATB1-shRNA2, and SATB1-shRNA3 cells was determined by MTT assay. (C) Apoptosis of SATB1-shRNA1 and empty vector control cells was determined by flow cytometry analysis of PI and Annexin V staining. (D) Flow cytometry analysis of cell cycle in empty vector control, SATB1-shRNA1, and SATB1-shRNA2 cells. (E) Soft agar colony formation of empty vector control, SATB1-shRNA1, SATB1-shRNA2, and SATB1-shRNA3 cells after 3 weeks. *p<0.001. (F) Migration of empty vector control, SATB1-shRNA1, SATB1-shRNA2, and SATB1-shRNA3 cells was determined by wound healing assay (left panel) and transwell migration assay (top right panel). The number of migrated cells in the transwell migration assay was quantified by counting the cells on the underside of the filter membrane in 5 randomly selected microscopic fields (lower right panel). *p<0.001, number of migrated cells compared to control. (G) Cell invasion of empty vector control, SATB1-shRNA1, SATB1-shRNA2, and SATB1-shRNA3 cells was determined by Matrigel transwell invasion assay. The number of invaded cells was quantified by counting cells on the underside of the filter membrane in 5 randomly selected microscopic fields. *p<0.001, number of invaded cells compared to control.

To evaluate the role of SATB1 in CRC progression, three stable SATB1-knockdown LoVo cell lines were established using shRNA. SATB1-shRNA1, SATB1-shRNA2, and SATB1-shRNA3 cell lines were effective in downregulating SATB1 mRNA and protein expression (Fig. 2A). Protein expression of SATB1 was barely detectable in SATB1-shRNA1 and SATB1-shRNA2 cells. SATB1 mRNA expression was reduced by almost 90% in SATB1-shRNA1 cells, and by 70% and 60% in SATB1-shRNA2 and SATB1-shRNA3 cells, respectively (Fig. 2A). The growth rate of all SATB1-shRNA cell lines was slower than that of the empty vector control cells (p<0.05, Fig. 2B). As shown in Fig. 2C, SATB1-shRNA1 cells had a significantly higher percentage of apoptotic cells than the control cells (45.1% v.s. 20.3%, p<0.01). However, the percentage of apoptotic cells was not significantly different between the SATB1-shRNA2, SATB1-shRNA3, and control cells (data not shown). SATB1 knockdown induced G1-phase cell cycle arrest: the percentage of cells in G0/G1 phase was higher in SATB1-shRNA1 (46.4%) and SATB1-shRNA2 cells (45.6%) than in the control cells (36.8%) (p<0.01; Fig. 2D). In contrast, the percentage of cells in G2/M and S phases was significantly lower in shRNA1 and shRNA2 cells than in the control cells (p<0.01; Fig. 2D). Cell cycle changes were not observed in SATB1-shRNA3 cells (data not shown). These findings indicated that SATB1 may play an important role in promoting the growth and survival of CRC cells.

Knockdown of SATB1 expression decreased the colony formation rate and colony size (Fig. 2E). In the control cells, almost 100 to 120 colonies could be observed in randomly selected microscopic fields. In contrast, the colony formation were much fewer in the three SATB1-shRNA cells (shRNA1: 28.75±8.5; shRNA2: 34.5±7.8; shRNA3: 40.5±4.5). Knockdown of SATB1 also reduced cell motility as evidenced by the wider wound closures in the 3 SATB1-shRNA cell lines in comparison to those in the control cells (Fig. 2F). The invasive capability was significantly lower in SATB1-shRNA1, shRNA2, and shRNA3 cells than in the control cells (p<0.001; Fig. 2G). These results were consistent with those of the transwell migration assay (Fig. 2F).

**Figure 3:**
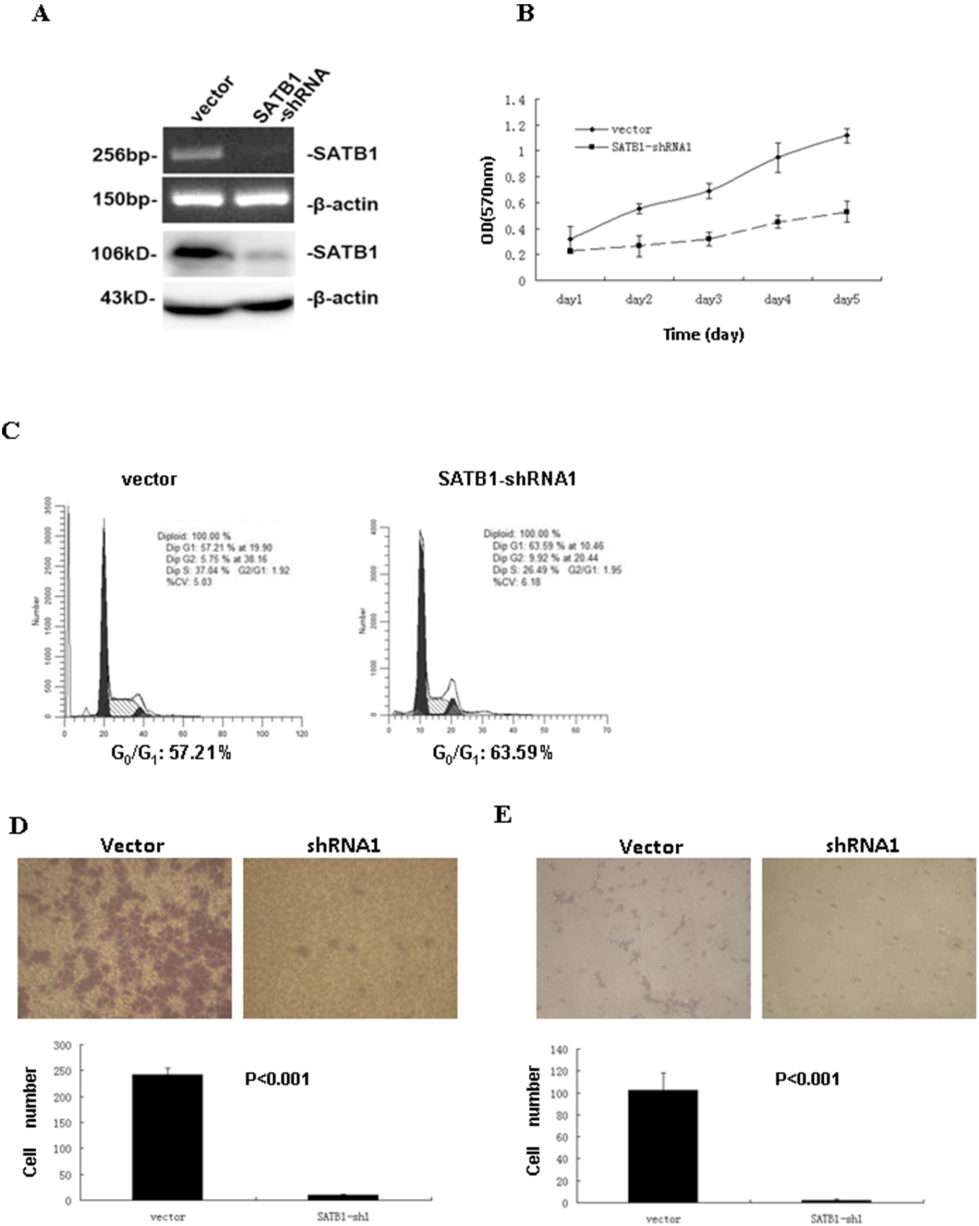
In RKO cell lines, knockdown of SATB1 expression reduces cell proliferation, migration, and invasion in vitro. (A) SATB1-knockdown RKO cell lines was established using shRNA1. Cells transfected with empty vector served as controls. The efficacy of SATB1 knockdown was verified by RT-PCR and western blot. β-actin was used to ensure equal loading. (B) Cell proliferation of empty vector control, and SATB1-shRNA1 RKO cells was determined by MTT assay. (C) Flow cytometry analysis of cell cycle in empty vector control, SATB1-shRNA1 RKO cells. (D) Migration of empty vector control, SATB1-shRNA1 RKO cells was determined by transwell migration assay. *p<0.001, number of migrated cells compared to control. (E) Cell invasion of empty vector control, SATB1-shRNA1 RKO cells was determined by Matrigel transwell invasion assay. *p<0.001, number of invaded cells compared to control.

Furthermore, we tested the growth rate and migration/invasion ability in RKO cell lines. SATB1-knockdown of RKO cell lines was established using shRNA1 (named SATB1-shRNA1 RKO cell lines). Both of the mRNA and protein levels of SATB1 were effectively downregulated by shRNA1 (Fig. 3A). Consistent with SATB1 knockdown experiments in LoVo cell lines, the growth rate of SATB1-shRNA1 RKO cell lines was slower than that of the empty vector control cells (p<0.05, Fig. 3B), and SATB1-shRNA1 RKO cell lines also showed the G1-phase cell cycle arrest (Fig. 3C). Migration assay provided the evidence that knockdown of SATB1 gene could reduce the cell motility (p<0.001 Fig. 3D). And in RKO cell lines, the invasive capability was significantly reduced by down-regulating SATB1 expression, as shown in the Fig. 3E (p<0.001).

### 3.4 Overexpression of SATB1 Promotes Cell Proliferation, Colony Formation, Migration, and Invasion in Vitro

**Figure 4:**
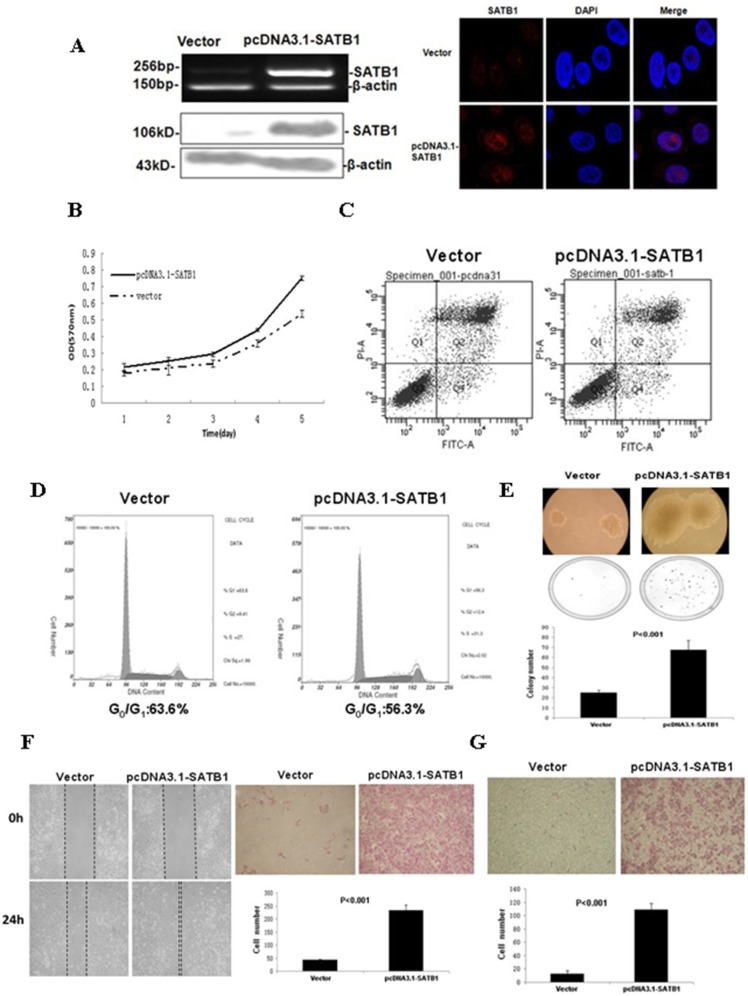
In SW480 cell lines, overexpression of SATB1 promotes cell proliferation, colony formation, migration, and invasion in vitro. (A) SW480 cells were transfected with the pcDNA3.1-SATB1 expression vector or empty vector. Upregulation of SATB1 mRNA and protein expression was verified by RT-PCR (top left panel), western blot (bottom left panel), and immunofluorescence (right panel). β-actin was used to ensure equal loading. (B) Cell proliferation of empty vector control and pcDNA3.1-SATB1 cells was determined by MTT assay. (C) Apoptosis of empty vector control and pcDNA3.1-SATB1 cells was determined by flow cytometry analysis of PI and Annexin V staining. (D) Flow cytometry analysis of cell cycle in empty vector control and pcDNA3.1-SATB1 cells. (E) Soft agar colony formation of empty vector control and pcDNA3.1-SATB1 cells after 3 weeks. (F) Migration of empty vector control and pcDNA3.1-SATB1 cells was determined by wound healing assay (left panel) and transwell migration assay (right panel). *p<0.001, number of migrated cells compared to controls. (G) Cell invasion of empty vector control and pcDNA3.1-SATB1 cells was determined by Matrigel transwell invasion assay (*p<0.001).

To further verify the role of SATB1 in CRC progression, an SATB1-overexpressing cell line was established using poorly metastatic SW480 cells. SATB1 overexpression was verified by western blot, RT-PCR, and immunofluorescence staining (Fig. 4A). The growth rate was higher in SATB1-overexpressing cells than in the empty vector control cells (p<0.05; Fig. 4B). The percentage of apoptotic cells was lower in the SATB1-overexpressing cells than that in the control cells (28.7% v.s. 36.1%, p<0.01; Fig. 4C). Overexpression of SATB1 promoted cell cycle progression from G0/G1 phase to G2/M and S phases (56.3% G0/G1 phase in SATB1-overexpressing cell lines versus 63.6% G0/G1 phase in control cells) (Fig. 4D). Colony formation rate and colony size were higher in the SATB1-overexpressing cells (67.5±9.5) than in the control cells (24.75±2.75) (Fig. 4E). SATB1 overexpression increased cell migration as evidenced by the complete wound closure and more migrated cells in SATB1-overexpressing cells (Fig. 4F). In addition, invasion capability was higher in the SATB1-overexpressing cells (109±8.7) than in the control cells (12.75±4.5) (Fig. 4G).

### 3.5 SATB1 Promotes the Growth and Metastasis of CRC Cells in Vivo

**Figure 5:**
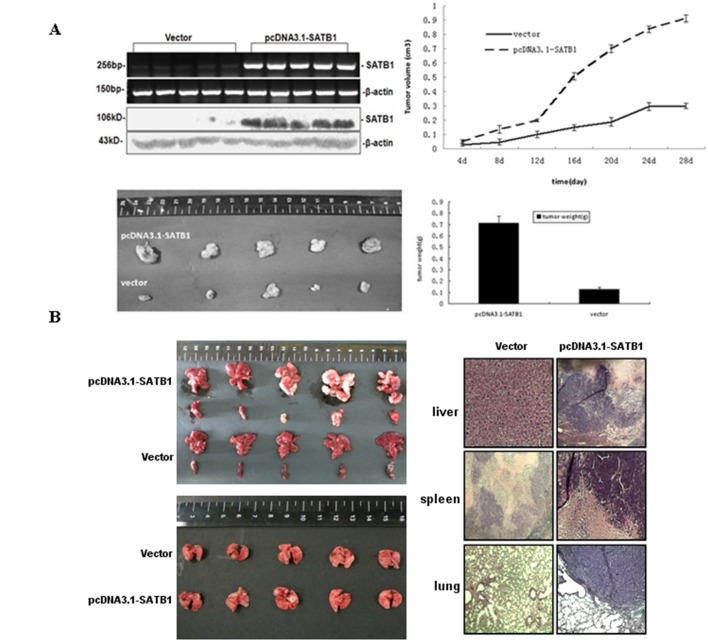
Overexpression of SATB1 promotes tumor growth and metastasis in vitro. (A) mRNA and protein expressions of SATB1 inxenograft tumors from empty vector control and pcDNA3.1-SATB1 cells were determined by RT-PCR and western blot, respectively (top left panel). Then, tumor growth was determined in an in vivo xenograft model. Stable SATB1-overexpressing and empty vector control cells (2×10^6^) were injected subcutaneously into the left and right dorsal flank, respectively, in 5 female BALB/c athymic mice. Tumor sizes were measured twice weekly, and the mice were sacrificed after 4 weeks. Tumor growth in the mice injected with empty vector control and pcDNA3.1-SATB1 tumor cells was determined by growth rate assay (top right panel). Tumor weights (bottom right panel) and photographs of tumors (bottom left panel) from mice injected with empty vector control and pcDNA3.1-SATB1 cells. *p<0.05, tumor weight compared to control. (B) Empty vector and pcDNA3.1-SATB1 cells were injected into the spleen and lateral tail vein of BALB/c athymic mice in CRC liver and lung metastasis models, respectively (n = 5 per treatment group). Photographs of tumor formation in the liver, spleen, and lung from mice injected with empty vector control and pcDNA3.1-SATB1 cells (left panel). Representative hematoxylin and eosin stains of the liver, spleen, and lung from mice injected with control vector cells and pcDNA3.1-SATB1 cells (right panel).

Next, we evaluated the effect of SATB1 overexpression on CRC carcinogenesis in vivo, the mRNA and protein expression of SATB1 were higher in tumors from the SATB1-overexpressing cells than in tumors from the empty vector cells (Fig. 5A). Tumors from the SATB1-overexpressing cells had a faster growth rate than tumors from the control cells, which was consistent with in vitro growth rates (p<0.01, Fig. 5A, top panel). The tumor weights from the SATB1-overexpressing cells were approximately 5–6 times heavier than those from the empty vector cells, indicating that SATB1 could promote the growth of colorectal tumors in vivo (Fig. 5A, bottom panel).

We also investigated the effect of SATB1 expression on the metastasis potential of CRC cells in vivo. Tumor formation in the liver and spleen was significantly promoted by SATB1 expression in CRC cells (Fig. 5B). Of the 5 mice in the SATB1-overexpressing cell group, 4 mice developed liver metastasis, whereas none of the mice in the control group developed liver metastasis (p = 0.01). Spleen tumor weight increase caused by SATB1 expression was non-significant (Fig. 5B). The spleen tumor weights weighed 0.242±0.1 g and 0.146±0.03 g in the SATB1-overexpressing group and the empty vector control group, respectively. Lung metastases developed in 3 of the 5 mice in the SATB1-overexpressing group, whereas lung metastasis was not observed in the empty vector control group (p = 0.038; Fig. 5B). These results indicated that SATB1 was sufficient to promote the metastasis of CRC.

### 3.6 SATB1 Regulates Gene Expression in CRC Cells

**Figure 6:**
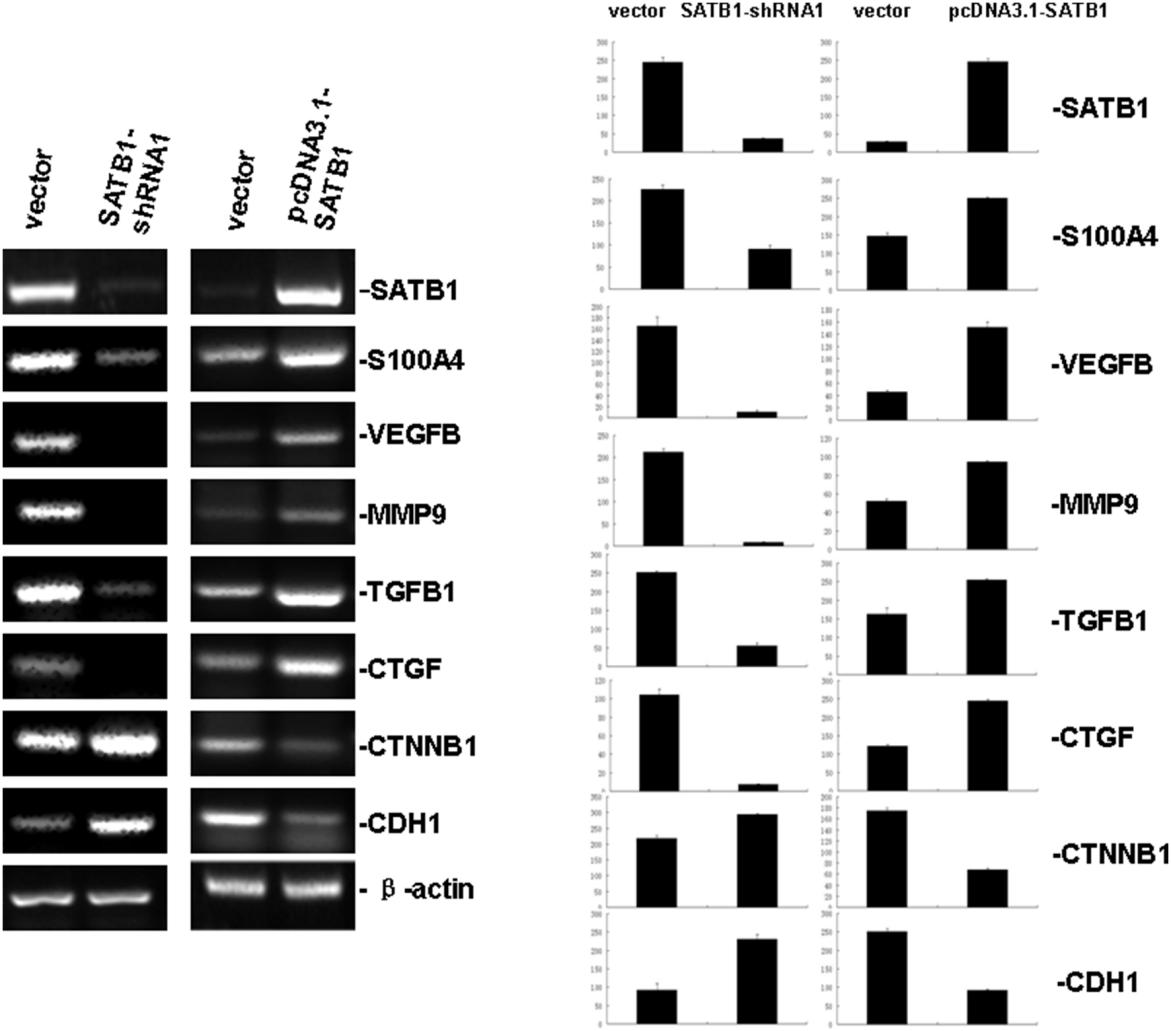
SATB1 regulates gene expression in CRC cells. The expression level of genes associated with CRC carcinogenesis and metastasis were determined in empty vector, SATB1-shRNA1, and pcDNA3.1-SATB1 cell lines by RT-PCR. Knockdown of SATB1 expression downregulated S100A4, VEGF-B, MMP-9, TGFβ1, and CTGF expression and upregulated CTNNB1 and CDH1 expression. SATB1 overexpression resulted in the opposite effect.

We evaluated the effect of SATB1 on the expression of genes associated with CRC carcinogenesis, invasion, and metastasis. The expression of S100A4, vascular endothelial growth factor-B (VEGF-B), matrix metalloproteinase-9 (MMP-9), transforming growth factor β1 (TGFβ1), and connective tissue growth factor (CTGF) was upregulated by SATB1 (Fig. 6). CTNNB1 (β-catenin gene) and CDH1 (E-cadherin gene) are crucial components of cell-cell adhesion complexes and their loss has been associated with tumor metastasis and poor clinical outcome. Both of these genes were downregulated by SATB1. Thus, SATB1 may promote acquisition of a more aggressive phenotype in CRC by significantly altering gene expression.

### 3.7 Demographics of CRC patients

Samples from a total of 560 patients were included in the tissue microarray. Detailed clinicopathological information was obtained from 520 patients, including 311 males and 209 females. A summary of the clinicopathological characteristics of the patients is shown in Table 1. The age of the patients at the time of surgery ranged from 21.9 to 86.2 years (median age = 58.8 years). According to TNM classification, 187 and 333 patients were classified as stage I−II and stage III−IV, respectively. The number of patients with right-sided tumors, left-sided tumors, middle-sided tumors, and rectal tumors was 182, 179, 25, and 134, respectively.

**Table 1:** Summary of patient characteristics (n = 520).

Clinicopathological features	No. of patients	(%) of patients
**Gender**		
Male	311	59.8
Female	209	40.2
**Age (years)**		
<60	254	48.8
≥60	266	51.2
**Tumor site**		
Right-sided	182	35.0
Left-sided	179	34.4
Middle-sided	25	4.8
Rectum	134	25.8
**Differentiation**		
Well-moderate	376	72.3
Poor	144	27.7
**Tumor size**		
<5 cm	317	61.0
≥5 cm	203	39.0
**Depth of invasion**		
T1+ T2	131	25.2
T3+ T4	389	74.8
**Lymph node metastasis**		
N0	231	44.4
N1–2	289	55.6
**Distant metastasis**		
M0	241	46.3
M1	279	53.7
**Vascular invasion**		
Absent	345	66.3
Present	175	33.7
**TNM stage**		
I + II	187	36.0
III + IV	333	64.0

### 3.8 SATB1 Expression is higher in CRC Tissues than in Non-Cancerous Tissues

**Figure 7:**
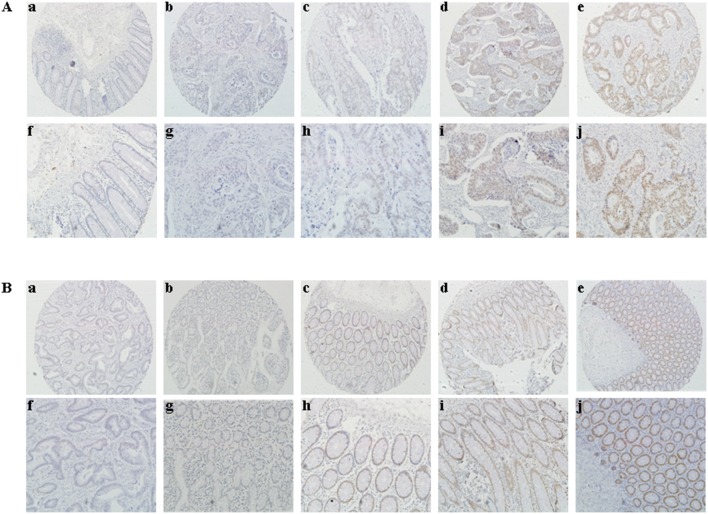
Representative immunohistochemical staining of SATB1 and SATB2 in colorectal cancer and matched normal mucosa. (A) Images (×100) representing immunohistochemical staining of SATB1 in (a) normal mucosa and colorectal cancer, ranging from (b) negative (c) weakly positive (d) moderately positive (e) strongly positive nuclear intensity. (f)/(g)/(h)/(i)/(j): Higher magnifications of panel a/b/c/d/e (magnification ×200). (B) Images (×100) representing immunohistochemical staining of SATB2 in (a) colorectal cancer and normal mucosa, ranging from (b) negative (c) weakly positive (d) moderately positive (e) strongly positive nuclear intensity. (f)/(g)/(h)/(i)/(j): Higher magnifications of panel a/b/c/d/e (magnification ×200).

Tissue microarrays constructed from 560 CRC cases were used to analyze SATB1 expression in cancerous tissues and matched non-cancerous mucosa. Nuclear staining of SATB1 protein was significantly higher in CRC tissues than in normal mucosa tissues (Fig. 7A). SATB1 expression in CRC tissues and normal mucosa tissues was 43.0% and 1.8%, respectively (p<0.001).

### 3.9 SATB2 Expression in CRC and its Association with SATB1 expression

SATB2 expression was tested in 551 CRC cases. Immunohistochemical staining showed that SATB2 protein was predominantly localized on Nucleus (Fig. 7B). SATB2 expression was markedly higher in normal mucosa than in carcinoma tissues (84.8% v.s. 46.9%, p<0.001). In 520 of these patients, both SATB1 and SATB2 expression were tested. SATB2 expression was significantly associated with SATB1 (p<0.001), as shown in Table 2.

**Table 2:** Association of SATB1 expression with clinicopathological features and SATB2 (n = 520).

Clinicopathological Features	SATB1 expressi		P
	Positive (%)	Negative (%)	
**Gender**			
Male	126 (40.5)	185 (59.5)	0.534
Female	79 (37.8)	130 (62.2)	
**Age (years)**			
<60	101 (39.8)	153 (60.2)	0.877
≥60	104 (39.1)	162 (60.9)	
**Tumor site**			
Right-sided	72 (39.6)	110 (60.4)	0.891
Left-sided	71 (39.7)	108 (60.3)	
Middle-sided	8 (32.0)	17 (68.0)	
Rectum	**54 (40.3)**	**80 (59.7)**	
**Differentiation**			
Well-moderate	124 (33.0)	252 (67.0)	<0.001
Poor	**81 (56.25)**	**63 (43.75)**	
**Tumor size**			
<5 cm	128 (40.4)	189 (59.6)	0.577
≥5 cm	77 (37.9)	126 (62.1)	
**Depth of invasion**			
T1+ T2	39 (29.8)	92 (70.2)	0.009
T3+ T4	166 (42.7)	223 (57.3)	
**Lymph node metastasis**			
N0	92 (39.8)	139 (60.1)	0.866
N1–2	113 (39.1)	176 (60.9)	
**Distant metastasis**			
M0	80 (33.2)	161 (66.8)	0.007
M1	125 (44.8)	154 (55.2)	
**Vascular invasion**			
Absent	136 (39.4)	209 (60.6)	0.999
Present	69 (39.4)	106 (60.6)	
**TNM stage**			
I + II	62 (33.2)	125 (66.8)	0.028
III + IV	143 (42.9)	190 (57.1)	
**SATB2**			
Positive	119(50.4)	117(49.6)	<0.001
Negative	86(30.3)	198(69.7)	

### 3.10 SATB1 Expression was Associated with Poor Overall Survival and Unfavorable Prognostic Factors

**Figure 8:**
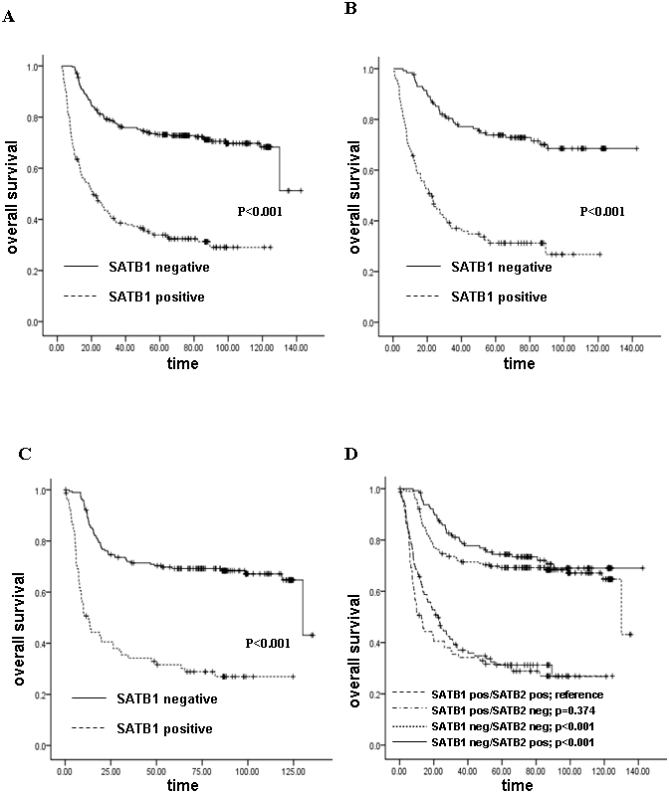
Kaplan-Meier estimates of the prognostic value of SATB1 expression. Kaplan-Meier survival analysis according to SATB1 expression in (A) all patients, (B) patients with SATB2 positive expression, (C) patients with SATB2 negative expression. (D) Kaplan-Meier estimates of colorectal cancer specific survival according to combinations of negative and positive SATB1 and SATB2 expression.

Detailed results of the association of SATB1 expression with clinicopathological features are shown in Table 2. SATB1 expression was markedly higher in patients with poorly differentiated tumors (p<0.001), higher invasion depth (p = 0.009), distant metastasis (p = 0.007), and advanced TNM stage (p = 0.028). SATB1 expression was not significantly correlated with other clinicopathological features including tumor site, tumor size, and lymph node metastasis. Kaplan-Meier survival analysis and log-rank test revealed a significant correlation between high SATB1 expression level and shorter survival time (p<0.001; Fig. 8A). The median survival time was 16.07±3.36 months for SATB1-positive patients and 74.67±3.87 months for SATB1-negative patients. Cox multivariate analysis showed that tumor differentiation, depth of invasion, lymph node metastasis, distant metastasis, and SATB1 expression were independent prognostic predictors of CRC (Table 3). Furthermore, we also tested the prognostic impact of SATB1 expression on patients with SATB2 positive or negative expression, respectively, and found that SATB1 expression was significantly associated with shorter survival time no matter in SATB2 positive patients or in SATB2 negative patients (p<0.001; Fig. 8B, C). And in CRC patients of our research, SATB2 expression was not an independent prognostic predictor (p = 0.836).

**Table 3:** Cox proportional hazard regression model analysis.

Variables	Multivariate analysis		
	Relative Risk	95% CI	P
**Differentiation**	0.632	0.447–0.836	0.001
**Depth of invasion**	4.877	2.575–9.238	<0.001
**Lymph node metastasis**	1.596	1.128–2.259	0.008
**Distant metastasis**	18.758	11.448–30.734	<0.001
**SATB1 expression**	1.446	1.094–1.910	0.009

Next, we analyzed the combined impact of SATB1 and SATB2 expression on prognosis of CRC patients. Results indicated that survival of patients in the SATB1-negative/SATB2-positive group was significantly better than that of patients with SATB1-positive/SATB2-negative or SATB1-positive/SATB2-positive expression (p<0.001; Fig. 8D). Although the median survival time of SATB1-negative/SATB2-positive group (74.47±3.70 months) was a little longer than that of SATB1-negative/SATB2-negative group (68.81±2.89 months), the difference of these two groups was not significant (p = 0.275). Similarly, there was not marked difference between survival time of SATB1-positive/SATB2-negative group and SATB1-positive/SATB2-positve group (the median survival time was 10.60±3.56 months and 21.07±3.21 months, respectively, p = 0.374).

## Discussion

SATB1, a tissue-specific nuclear matrix-attachment DNA-binding protein, is involved in chromatin structure packaging and gene expression [16]. Initial studies about SATB1 focused on its role in immune cells, in particular its association with T-cell development [6], [17]–[19]. In 2008, Han et al. [11] reported that SATB1 expression promoted the growth and metastasis of breast cancer. Furthermore, SATB1 was found to have a high prognostic significance in breast cancer, independent of lymph node status. Recently, SATB1 expression has been shown to correlate with unfavorable tumor characteristics in rectal cancer [20]. However, limited data is available on the role of SATB1 expression in CRC development and progression. A recent report showed that SATB1 expression in CRC was significantly associated with β-catenin overexpression and microsatellite stability [21]. And recently, Zhang et al [22] and Fang et al [23] have shown some malignant features related to SATB1. In the present study, we demonstrated the involvement of SATB1 in CRC growth and metastasis in vitro and in vivo, more intensively. In addition, we also evaluated the expression of SATB2 in CRC and its impact on prognosis of CRC patients.

It has been shown that SATB1 was overexpressed in a variety of tumor types including breast cancer [11], laryngeal squamous cell carcinoma [12], gastric cancer [13], [14], and malignant cutaneous melanoma [15]. In this study, we found that SATB1 mRNA and protein were highly expressed in metastatic CRC cell lines and CRC tumors and corresponding hepatic metastasis foci. Additionally, SATB1 mRNA and protein were increased in poorly differentiated CRC tumors in comparison to moderately differentiated CRC tumors. These data suggested that the SATB1 gene may be associated with CRC progression and metastasis. To further investigate the role of SATB1 in CRC, we evaluated the effect of SATB1 expression on CRC growth and metastasis in vitro and in vivo using stable SATB1-overexpressing and SATB1-knockdown cell lines. And in order to avoid possible specific phenomena in experiments, parallel knockdown experiments by using LoVo cell lines and RKO cell lines were carried out. The results showed that knockdown of SATB1 expression could reduce cell proliferation, colony formation, migration, and invasion. In contrast, SATB1 overexpression promoted the acquisition of an aggressive phenotype in CRC that was associated with a high rate of cell proliferation, colony formation, migration, and invasion. SATB1 promoted cell survival by inhibiting apoptosis and promoting G0/G1-phase progression into G2/M and S phases. SATB1 also promoted the growth and metastasis of CRC cells in vivo.

SATB1 has been shown to promote tumor growth and metastasis by altering gene profiles [11]. SATB1 expression was shown to markedly alter the expression of over 1000 breast cancer genes including metastasis-associated genes and tumor suppressor genes [11]. In Björn Nodin’s research [21], SATB1 has been shown to interact with beta-catenin and recruit it to its genomic binding sites. In our research, we found that SATB1 upregulated the expression of multiple genes associated with migration, invasion, angiogenesis, and metastasis including the small calcium binding protein S100A4 [24], [25], VEGF-B [26]–[28], the type IV collagenase MMP-9 [29], [30], the cytokine TGFβ1 [31]–[33], and CTGF [34]–[36]. CTNNB1 and CDH1 are crucial components of cell-cell adhesion complexes, and their loss has often been associated with tumor metastasis and poor clinical outcome [37], [38]. Interestingly, in Björn Nodin’s research [21], SATB1 expression was found to be significantly associated with beta-catenin (CTNNB1) overexpression. However, in our research, both CTNNB1 and CDH1 were downregulated by SATB1. There might be many complex factors affecting the interaction between CTNNB1 and SATB1 [21], [39]. Our study has only reported the expression level of CTNNB1 and SATB1 in the CRC cell lines in vitro. However, in vivo, in view of complex factors that might affect the interaction between these two proteins, the actual function of CTNNB1 in the progression of CRC and its relationship with SATB1 needs further research. Above all, SATB1 expression may promote the acquisition of an aggressive phenotype in CRC by inducing significant modification of the gene expression patterns in CRC cells.

Recently, two studies have focused on the relationship of SATB1 with colorectal cancer. In one study, Zhang et al [22] showed over-expression of SATB1 in 80 CRC cases, and found that SATB1 was associated with tumor differentiation and pTNM stage. And they further investigated the effect of down regulation of SATB1 expression on malignant phenotypic features in vitro. In the other study, Fang et al [23] also assessed SATB1 expression in 30 CRC samples, and by enhancing expression of SATB1, some phenotypic features were investigated in vitro and in vivo. In our research, more abundant data and more strong evidence were provided. As above-mentioned findings, six colorectal cell lines and a huge sample of 560 CRC cases were taken into account in our study. SATB1 expression was significantly correlated with unfavorable prognostic markers including poor differentiation, higher invasion depth, presence of distant metastasis, and advanced TNM stage. In breast cancer [11], SATB1 expression was found not restricted to advanced clinical stages of disease, and the SATB1 level had high prognostic significance in breast cancer, independent of the lymph node status. In Björn Nodin’s research [21], SATB1 expression was shown to be a factor of poor prognosis in SATB2 negative tumors. In our research, SATB1 expression was correlated with poor prognosis either in SATB2-positive CRC patients or in SATB2-negative CRC patients. Cox multivariate analysis showed that SATB1 was a negative independent predictor of overall survival in CRC patients. Histological grade, depth of invasion, nodal stage, and distant metastasis stage were also independent predictors of overall survival. Thus, assessment of SATB1 expression in combination with these independent prognostic factors may predict patient outcome in CRC more accurately.

Remarkably, we have also evaluated SATB2 expression in CRC and its relationship with SATB1 or prognosis in CRC patients. Special AT-rich sequence-binding protein 2 (SATB2), a nuclear matrix associated protein, is a close homologue to SATB1 [40]. In our research, we found that SATB2 was more abundantly expressed in non-cancerous mucosa than in cancerous tissue, which is in line with previous findings by Eberhard, J.et al [41]. And a strong association was observed between SATB1 and SATB2 expression. In some studies, immunohistochemical assay showed that loss expression of SATB2 was associated with poor prognosis in CRC [41], [42]. And another study proclaimed prognostically antagonistic effect of SATB1 and SATB2 [21]. However, in our study, Kaplan-Meier analysis did not reveal prognostic significance of SATB2 expression in CRC patients (p = 0.836). Kaplan-Meier estimates of colorectal cancer-specific survival according to combination of SATB1 and SATB2 expression showed that SATB1-negative/SATB2-positive and SATB1-negative/SATB2-negative groups have the better survival time. The SATB1-positive/SATB2-positive and SATB1-positive/SATB2-negative groups have the worse survival time. There is no marked difference between the survival time of SATB1-negative/SATB2-positive and SATB1-negative/SATB2-negative groups or between that of SATB1-positive/SATB2-positive and SATB1-positive/SATB2-negative groups. Our results indicated that the prognostic value of SATB1 did not differ according to SATB2 expression.

Strikingly, in this study, the effects of up- and down-regulation of SATB1 expression on malignant phenotypic features in colorectal cancer cells were both investigated. Based on more evidence, we could exactly pronounce that SATB1 could promote cell proliferation, colony formation and invasion in vitro. Furthermore, besides the xenograft animal model of tumorigenesis, lung and liver metastasis model were used to further verify the effect of SATB1 on metastasis potential of CRC cells in vivo. Consistent with the findings from in vitro experiments, the function of SATB1 to promote the tumorigenesis and metastasis was further confirmed. Based on our assessment, SATB1 has been shown to be significantly involved in mechanism of CRC carcinogenesis and metastasis. And gene expression pattern in CRC cells might be regulated by SATB1, so further mechanism exploration will be carried out.

Above all, our findings indicated an important role of SATB1 in CRC carcinogenesis and metastasis. Therefore, SATB1 may represent an important prognostic biomarker and therapeutic target for CRC.
